# High HCV seroprevalence and HIV drug use risk behaviors among injection drug users in Pakistan

**DOI:** 10.1186/1477-7517-3-26

**Published:** 2006-08-16

**Authors:** Irene Kuo, Salman ul-Hasan, Noya Galai, David L Thomas, Tariq Zafar, Mohammad A Ahmed, Steffanie A Strathdee

**Affiliations:** 1 Department of Mental Health, Johns Hopkins Bloomberg School of Public Health, Baltimore, Maryland 21205, USA; 2 Nai Zindagi, Office No. 37-38, Top floor Beverly Center, Jinnah Avenue, Blue Area, Islamabad, Pakistan; 3 Department of Epidemiology, Johns Hopkins Bloomberg School of Public Health, Baltimore, Maryland 21205, USA; 4 Division of Infectious Diseases, Johns Hopkins School of Medicine, Baltimore, Maryland 21205, USA; 5 Division of International Health and Cross Cultural Medicine, University of California at San Diego, La Jolla, California 92093, USA

## Abstract

**Introduction:**

HIV and HCV risk behaviors among injection drug users (IDUs) in two urban areas in Pakistan were identified.

**Methods:**

From May to June 2003, 351 IDUs recruited in harm-reduction drop-in centers operated by a national non-governmental organization in Lahore (Punjab province) and Quetta (Balochistan province) completed an interviewer-administered survey and were tested for HIV and HCV. Multivariable logistic regression identified correlates of seropositivity, stratifying by site. All study participants provided written, informed consent.

**Results:**

All but two were male; median age was 35 and <50% had any formal education. None were HIV-positive; HCV seroprevalence was 88%. HIV awareness was relatively high, but HCV awareness was low (19%). Injection behaviors and percutaneous exposures such as drawing blood into a syringe while injecting ('jerking'), longer duration of injection, and receiving a street barber shave were significantly associated with HCV seropositivity.

**Discussion:**

Despite no HIV cases, overall HCV prevalence was very high, signaling the potential for a future HIV epidemic among IDUs across Pakistan. Programs to increase needle exchange, drug treatment and HIV and HCV awareness should be implemented immediately.

## Background

Until recently, Pakistan had been classified as a country with a low seroprevalence but high potential for a HIV epidemic [1,2]. Several reasons given for this included the lack of resources to screen donations at blood banks, the use of unsterilized medical equipment, and the high prevalence of unnecessary medical injections where needles and syringes are often reused without proper sterilization [1,3].

A growing risk for the transmission of bloodborne diseases in Pakistan is related to injection drug use [2]. Recent global HIV outbreaks in Indonesia, China, Vietnam, Eastern Europe and Central Asia have been driven by injection drug use [2,4-7]. Pakistan is especially vulnerable because it is a main trafficking route for opiates smuggled from Afghanistan, the world's largest producer of opium [8]. A recent report by the United Nations estimated a country-wide annual prevalence of 0.8% of opiate use in Pakistan, compared with 0.4% in India and 0.6% in the United States [9]. Moreover, a 2002 report indicated that of the 500,000 heroin users estimated at that time, 60,000 were thought to be injectors [10], although accurate estimates are lacking.

Of late, overall HIV prevalence had remained very low in drug using populations (0% to 2%) [11,12] and among commercial sex workers and prisoners, where seroprevalence rates have ranged from 0% to 1.8%, in Karachi, Pakistan's most populous city situated in the southern Sindh province [3,12]. To our knowledge, no estimates of HIV infection have been published from Balochistan, the western region bordering Afghanistan.

However, in 2003, HIV/AIDS officials in Sindh reported an outbreak of HIV infection among injection drug users (IDUs) in a prison located outside of Karachi, in which among 175 prisoners tested, 17 (9.7%) were HIV seropositive [13]. In 2004, an outbreak of HIV among injection drug users was detected in Karachi, where 23% of IDUs tested were HIV positive [2], compared to only one documented HIV-positive case in the previous seven months in the same study population [14]. However, HIV prevalence rates among drug users in other regions of Pakistan have been seldom reported or remain unpublished.

To date, few studies have focused on hepatitis C virus (HCV) among drug users in Pakistan. One unpublished study of IDUs conducted in 1999 in Lahore (eastern Pakistan bordering India) revealed a HCV seroprevalence of 89% [11], compared to 6.5% seroprevalence found in the general population [15].

In light of a recent HIV outbreak in Karachi, we provide a report on the prevalence of HIV, HCV, and related risk behaviors among injection drug users in Lahore in the east and Quetta, which borders Afghanistan on the western border. The objective of this study was to determine baseline HIV and HCV seroprevalence and identify injection-related and percutaneous risk behaviors associated with seropositivity within a population of IDUs from these two regions in Pakistan. This study provides insight into the potential for future spread of these bloodborne diseases in other parts of Pakistan and sheds light on urgent areas for HIV prevention.

## Methods

### Study design and study population

A cross-sectional survey was conducted between May and June 2003 among IDUs attending two drop-in centers located in Lahore and in Quetta. The drop-in centers were operated by Nai Zindagi, a Pakistani non-governmental organization committed to the provision of drug treatment and harm reduction services to drug users.

The Lahore drop-in center was opened in July 2001 and is situated two blocks from Ali Park, a public space located in the red light district of Lahore where several hundreds of drug users congregate and sleep daily. The Quetta drop-in center was opened in early 2001 and, due to its close proximity to Afghanistan and Iran, is frequented by individuals of various nationalities including Afghans, Pakistanis (e.g., Pathans, Balochis), Tajiks and Iranians. Both drop-in centers provide free basic health and wound care, counseling, referrals to drug treatment, snacks and tea, bathing facilities and a relaxation room for clients.

All clients utilizing the drop-in centers were eligible for the study if they were 18 years of age or older and reported ever having injected heroin, morphine and/or other pharmaceutical drugs in their lifetime. All participants were read aloud the study consent form and provided written informed consent of enrollment, either as a signature or a thumbprint if illiterate.

### Data collection

Study participants completed a structured questionnaire based on instruments from previous studies [16-18]. The questionnaire was pilot-tested and was developed in English, translated into Urdu (the official language of Pakistan), and then independently back-translated to verify content validity. Because the local study population had such little previous exposure to computers, we were unable administer the questionnaire using audio-assisted computerized self-interview (ACASI); therefore, questionnaires were interviewer-administered.

Questionnaire data were entered into a computerized database (Microsoft Access), which was customized with built-in skip patterns and response limits to ensure high data quality. A random sample of 50 questionnaires from each study site was selected for double data entry. Discrepancies and systematic errors were reviewed and resolved by the data manager in Pakistan under the guidance of the research team from Johns Hopkins.

Major exposure categories considered as potential correlates of HIV and HCV seropositivity included: 1) drug use behaviors, such as the frequency and duration of injection drug use and sharing syringes; 2) medical and other percutaneous exposures, such as surgery, dental work, medical injections and receiving a barber shave; and 3) sexual behaviors, such as paying for sex and condom use. Participants were also asked about the practice of deliberately drawing blood into the syringe and re-injecting the blood-drug mixture (referred to locally as "jerking"; this is also known as "booting" or "registering" in other countries [19-21]).

Demographic information included variables such as age, education, marital status, income, and current and former employment. Participants who reported spending twenty-four hours a day on the streets were considered homeless. Income was categorized as earning less than 3,000 rupees (approximately $50 US) per month (or being "very poor") versus earning 3,000 rupees or more per month. HIV and HCV awareness was assessed through questions that asked if participants had ever heard of HIV or HCV before.

All participants provided a blood specimen at the study visit and received HIV and HCV pre-test counseling by trained interviewers and were compensated 200 rupees (equivalent to $4 US) for completing the study visit. Participants were asked to return to the study site after one month to receive their serology results and post-test counseling. The study was approved by the Johns Hopkins Bloomberg School of Public Health Committee on Human Research and Nai Zindagi's institutional review board.

### Serology

Blood specimens were tested for the presence of antibodies to HIV and HCV within 24 hours. Initial HIV antibody screening was conducted using ELISA (Thermo Labsystems). Samples testing negative initially for HIV were considered HIV-seronegative. Positive samples were re-confirmed by testing the sample in triplicate using two different ELISA tests (Thermo Labsystems and Vironostika/Organon Teknika) and a latex-based system (Capillus/Trinity Biotech) according to the manufacturers' instructions. Samples were considered HIV-seronegative if at least two of the three tests were negative and were considered HIV-seropositive only if all three tests were positive. Samples that were positive for two tests but not the third were considered indeterminate. All serological tests were run using positive and negative controls to ensure the quality of testing.

Initial screening for HCV was conducted using a third-generation ELISA test (BioChem ImmunoSystems/Adaltis). Samples found to be negative on the preliminary screen were considered HCV-seronegative. Initially positive and borderline samples were re-tested using the same assay. Samples were considered positive if the sample tested positive on the second run; samples testing positive on the first run and negative on the second run were considered indeterminate.

### Data analyses

Chi-square tests were used to compare categorical variables. All continuous variables, such as age and duration of drug use, were initially analyzed by comparing means or medians depending on their distribution using the Students t-test and Mann-Whitney test, respectively. Variables were then categorized based on their distribution to facilitate interpretation, with one exception: duration from initiation of injection was categorized based on a previous finding that the association of being HCV exposure rapidly increases after the initial year of injection [22]. In this case, to create a more stable variable, duration of injection drug use was dichotomized as ≤2 years versus >2 years duration of injection.

Univariate logistic regression analysis was used to identify potential correlates of HIV and HCV seropositivity. Variables attaining a p-value ≤ 0.10 were considered as potential correlates and were included in an initial multivariate model. In a manual fashion, stepwise multiple logistic regression using backwards elimination was used to identify independent associations of correlates of HIV and HCV seropositivity. Variables achieving a value of p ≤ 0.05 were retained in the final model.

Age, nationality, and site were examined for potential effect modification using interaction terms within models. Effect modification was considered to be present if the interaction term(s) attained a significance level of p ≤ 0.10. We found evidence of effect modification by site and income and duration of injection (main exposure variables); therefore all analyses subsequent were stratified by site. All data management and statistical analyses were conducted using STATA version 8.0 (College Station, Texas, U.S.A., 2003).

## Results

### Overview

A total of 351 IDUs were recruited; 255 (72.6%) were enrolled in Lahore and 96 (27.4%) were enrolled in Quetta. Table 1 displays demographic characteristics and comparisons by study site. All study participants from Lahore were male, while all but two from Quetta were male (97.9%). IDUs in Lahore were older, more likely to be Pakistani, unmarried, homeless, and very poor than in Quetta (p < 0.05). A significantly lower proportion of IDUs in Lahore than in Quetta had ever worked abroad outside of Pakistan (4.7% versus 19.8%, p < 0.001).

**Table 1:** Demographic characteristics of injection drug users (IDUs) in Lahore and Quetta, Pakistan.

Characteristic	Total N = 351 (%)	Lahore n = 255 (%)	Quetta n = 96 (%)	χ^2 ^p-value
Nationality				
Pakistani	331 (94.3)	254 (99.6)	77 (80.2)	
Afghan or Iranian	20 (5.7)	1 (0.4)	19 (19.8)	<0.001
				
Age (in years); mean (SD)	34.4 (8.9)	35.4 (8.5)	31 (9.2)	<0.001*
				
Any formal education				
No	197 (56.1)	141 (55.3)	56 (58.3)	
Yes	154 (43.9)	114 (44.7)	40 (41.7)	0.61
Marital status				
Single, divorced, widowed	281 (80.1)	39 (15.3)	31 (33.3)	
Married	70 (19.9)	216 (76.9)	65 (67.7)	<0.001
Currently employed				
No	244 (69.5)	180 (70.6)	64 (66.7)	
Yes	107 (30.5)	75 (19.1)	32 (33.3)	0.48
Being very poor (earned <3000 rupees/month)				
No	278 (79.2)	191 (74.9)	87 (90.6)	
Yes	73 (20.8)	64 (25.1)	9 (9.4)	0.001
Ever worked outside of Pakistan				
No	320 (91.2)	243 (95.3)	77 (80.2)	
Yes	31 (8.8)	12 (4.7)	19 (19.8)	<0.001
Homeless				
No	165 (47.0)	111 (43.5)	54 (56.3)	
Yes	186 (53.0)	144 (56.5)	42 (43.7)	0.03
HCV serostatus				
Negative	42 (12.0)	18 (7.1)	24 (25.0)	
Positive	309 (88.0)	237 (92.9)	72 (75.0)	<0.001
Ever heard of HCV before^†^				
No	283 (80.9)	194 (76.4)	89 (92.7)	
Yes	67 (19.1)	60 (23.6)	7 (7.3)	0.001
Ever heard of HIV before				
No	53 (15.1)	36 (14.1)	17 (17.7)	
Yes	298 (84.9)	219 (85.9)	79 (82.3)	0.40

* P-value based on t-test.

^† ^One response was missing; percentages based on available data.

SD = standard deviation

None were found to be HIV positive; however, HCV prevalence was very high and was significantly higher in Lahore than in Quetta (92.9% versus 75.0%, p < 0.001). Although most IDUs had heard of HIV (84.9%), only one-fifth had ever heard of HCV (19.1%). There was no significant difference in the level of HIV awareness in Quetta and Lahore, but a significantly higher proportion in Lahore knew about HCV than in Quetta (23.6% versus 7.3%, p = 0.001). While nearly all of the study participants returned to the drop-in center to receive services, only 6% of the study population requested to receive their HIV and HCV serology results.

### HIV and HCV risk behaviors

IDUs in Lahore versus Quetta had been using drugs longer (median 19 versus 14 years, respectively, p = 0.003) and had a longer injection history (median 7 vs. 3 years, p < 0.001). Table 2 displays HIV and HCV risk behaviors found in the study population and univariate analyses for HCV seropositivity. A significantly higher proportion of IDUs in Lahore versus Quetta had injected drugs in the past 6 months (97.3% versus 67.7%, p < 0.001), of whom most were injecting daily (89.9% versus 67.7%, p < 0.001). Among IDUs who did not inject in the past 6 months, the median time since last injection was 1 year (IQR: 1–2.4 years). The majority of IDUs in Lahore (91.0%) preferred injecting a combination of liquid buprenorphine, anti-histamine and tranquilizers. In Quetta, 58.3% preferred injecting heroin alone, while 41.7% injected heroin in combination with a liquid antihistamine and/or a tranquilizer.

**Table 2:** HIV and HCV risk behaviors and univariate analysis of potential correlates of HCV seropositivity among injection drug users (IDUs) in Pakistan, stratified by site.

	Lahore			Quetta		
Characteristic	Total n = 255	HCV+ (%)	OR (95% CI)	Total n = 96	HCV+ (%)	OR (95% CI)

Being very poor (<3000 rupees/month)						
No	191	177 (93)		87	68 (78)	
Yes	64	60 (94)	1.2 (0.4–3.7)	9	4 (44)	0.2 (0.1–0.9)^a^
Ever worked outside of Pakistan						
No	243	227 (93)		77	63 (82)	
Yes	12	10 (83)	0.4 (0.1–1.7)	19	9 (47)	0.2 (0.1–0.6)^a^
Currently homelessness						
No	111	98 (88)		54	38 (70)	
Yes	144	139 (97)	3.7 (1.3–10.7)^a^	42	34 (81)	1.8 (0.7–4.7)
						
Drug Use Behaviors						
Duration of injection drug use						
≤2 year	34	27 (79)		40	29 (73)	
2+ years	221	210 (95)	4.9 (1.8–13.8)^a^	56	43 (77)	1.3 (0.5–3.2)
Currently injecting (w/in last 6 months)						
No	7	6 (86)		31	19 (61)	
Yes	248	231 (93)	2.6 (0.3–19.9)	65	53 (82)	2.8 (1.1–7.3)^a^
Injected daily						
No	32	27 (84)		52	35 (67)	
Yes	223	210 (94)	3.0 (1.0–9.0)^a^	44	37 (84)	2.6 (1.0–6.9)^a^
Ever "jerked"*						
No	23	18 (78)		8	3 (38)	
Yes	232	219 (94)	4.7 (1.5–14.6)^a^	88	69 (78)	6.1 (1.3–27.6)^a^
Ever borrowed a needle or syringe						
No	82	75 (91)		35	22 (63)	
Yes	173	162 (94)	1.4 (0.5–3.7)	61	50 (82)	2.7 (1.0–6.9)^a^
Ever shared injection tools/ampoule						
No	69	61 (88)		36	22 (61)	
Yes	186	176 (95)	2.3 (0.9–6.1)^a^	60	50 (83)	3.2 (1.2–8.3)^a^
Always using a new syringe						
No	224	210 (94)		67	52 (78)	
Yes	31	27 (87)	0.5 (0.1–1.5)	29	20 (69)	0.6 (0.2–1.7)
						
Medical and Percutaneous Exposures						
Ever had any surgery						
No	146	137 (94)		63	47 (75)	
Yes	108	99 (92)	0.7 (0.3–1.9)	32	25 (78)	1.2 (0.4–3.3)
Ever received blood transfusion						
No	236	219 (93)		90	66 (73)	
Yes	19	18 (95)	1.4 (0.2–11.1)	6	6 (100)	--
Ever received medical injection**						
No	46	43 (93)		35	27 (77)	
Yes	209	194 (93)	0.9 (0.3–3.3)	61	45 (74)	0.8 (0.3–2.2)
Ever had any dental work						
No	145	134 (92)		59	45 (76)	
Yes	110	103 (94)	1.2 (0.5–3.2)	37	27 (73)	0.8 (0.3–2.2)
Ever received shave from barber						
No	3	2 (67)		8	4 (50)	
Yes	252	235 (93)	6.9 (0.6–80.1)	88	68 (77)	3.4 (0.8–14.8)^a^
Ever shared razor blade						
No	164	151 (92)		51	39 (76)	
Yes	90	85 (94)	1.5 (0.5–4.2)	44	33 (75)	0.9 (0.4–2.4)
Ever got a body piercing						
No	167	153 (92)		81	59 (73)	
Yes	88	84 (95)	1.9 (0.6–6.0)	15	13 (87)	2.4 (0.5–11.6)
Ever got a tattoo on body						
No	151	138 (91)		41	32 (78)	
Yes	104	99 (95)	1.9 (0.6–5.4)	55	40 (73)	0.8 (0.3–1.9)
						
Sexual History^†^						
Ever had sexually transmitted infection						
No	79	75 (95)		39	26 (67)	
Yes	153	141 (92)	0.6 (0.2–2.0)	46	37 (80)	2.1 (0.8–5.5)
Ever paid for sex						
No	76	71 (93)		25	20 (80)	
Yes	164	153 (93)	1.0 (0.3–2.9)	60	43 (72)	0.6 (0.2–2.0)
Ever had sex with a man or boy^‡^						
No	119	109 (92)		52	39 (75)	
Yes	121	115 (95)	1.8 (0.6–5.0)	31	23 (74)	1.0 (0.3–2.7)
Ever use a condom during sex						
No	150	141 (94)		73	57 (78)	
Yes	90	83 (92)	0.8 (0.3–2.1)	12	6 (50)	0.3 (0.1–1.0)

* Jerking refers to the practice of drawing blood into the syringe while injecting drugs.

** Includes professional and non-professional medical injections

^† ^Includes only those who have ever had sex (Lahore: n = 240; Quetta: n = 85); totals based on available data.

^‡^Two female participants were excluded from these analyses.

^a ^p ≤ 0.10

In both sites, 91.2% reported the practice of deliberately drawing their blood into the syringe when they injected drugs ("jerking"). Two-thirds reported ever borrowing a syringe/needle from someone else. A significantly higher proportion of IDUs in Lahore than in Quetta reported sharing injection tools (cotton/cloth, spoons, cookers and rinse water) and ampoules containing liquid drug preparations (72.9% versus 62.5%, p = 0.06). Conversely, only 12.2% in Lahore versus 30.2% in Quetta claimed to have always used a clean syringe every time they injected (p < 0.001).

Forty percent had ever undergone major or minor surgery, and 25 (7.1%) had ever received a blood transfusion, of whom nearly all were HCV-positive. The prevalence of receiving a medical injection from either a professional or non-professional (from an ayurvedic or 'quack') was higher in Quetta than Lahore (82.0% versus 63.5%, respectively, p < 0.001). Although most (96.9%) IDUs in both sites had received a barber shave, sharing razor blades was more common among participants in Quetta than Lahore (46.3% vs. 35.4%, p = 0.06). Blood donation (29.9% vs. 19.9%, p = 0.06) and body piercing (34.5% vs. 15.6%, p = 0.001) were more common in Lahore than Quetta, although tattooing was more prevalent in Quetta (57.3% versus 40.8%, p = 0.01).

Nearly all IDUs (92.6%) reported ever having sex, of whom a higher proportion in Lahore than Quetta reported ever having a STI (66.0% vs. 54.1%, p = 0.05). Most IDUs (68.9%) had ever paid for sex in their lifetime, but a significantly higher proportion of male IDUs in Lahore than Quetta ever had sex with a man or boy (50.4% vs. 37.4%, p = 0.04). Few had ever used a condom, although the proportion was higher in Lahore versus Quetta (37.5% vs. 14.2%, p < 0.001). Because of low risk of transmission of HCV via unprotected sex, we did not include sexual behaviors in the analyses of HCV correlates.

### Correlates of HCV seroprevalence

In Lahore, being homeless, having a longer duration of injection drug use (>2 years), injecting drugs daily, ever jerking, and sharing injection tools and ampoules were univariately associated with HCV seropositivity (Table 2). In Quetta, being very poor and having ever worked outside of Pakistan were negatively associated with HCV seropositivity on a univariate level. Also, being a current injector, ever having jerked, ever sharing syringes and sharing injection tools and ampoules and ever receiving a barber shave were positively associated with HCV seropositivity.

In multivariate analyses (Table 3), in Lahore, having ever jerked was independently associated with HCV seropositivity (adjusted odds ratio [AOR]: 3.4; 95% confidence interval [CI]: 1.0, 11.5), as was having injected for a longer duration (AOR: 4.3; 95% CI: 1.5, 12.6). Moreover, being currently homeless was also independently associated with higher odds of HCV seropositivity (AOR: 3.0; 95% CI: 1.0, 9.0).

**Table 3:** Multivariate model of correlates of HCV seropositivity among IDUs in Pakistan, stratified by site.

	Lahore (n = 255)		Quetta (n = 96)	
Variable	AOR	95% CI	AOR	95% CI
Currently homeless	3.0	1.0 – 9.0^a^	--	--
>2 years of injection drug use	4.3	1.5 – 12.6^a^	--	--
Ever "jerked"*	3.4	1.0 – 11.5^a^	7.3	1.3 – 41.4^a^
Ever received a shave by a barber	--	--	4.9	0.9 – 27.6
Ever worked outside of Pakistan	--	--	0.2	0.04 – 0.5^a^
Being very poor (<3000 rupees/mo)	--	--	0.2	0.03 – 0.9^a^

* Jerking refers to the practice of pumping blood in and out of the syringe while injecting drugs.

^a ^p ≤ 0.05

In Quetta, IDUs who had ever jerked had a seven-fold higher odds of HCV seropositivity than those who did not (AOR: 7.3; 95% CI: 1.3, 41.4). Although attaining only marginal statistical significance, injectors who had ever received a barber shave had a higher odds of HCV seropositivity compared to those who had not (AOR: 4.0; 95% CI: 0.9, 27.6). Having ever worked outside of Pakistan (AOR: 0.2; 95% CI: 0.04, 0.5) and being very poor (AOR: 0.2; 95% CI: 0.03, 0.9) were both negatively associated with HCV seropositivity in Quetta.

## Discussion

In this study of IDUs from two cities in Pakistan, there were no cases of HIV infection. However, a high prevalence of HCV seropositivity was observed among these IDUs (88%), consistent with other studies of adult IDU populations worldwide [11,23-25]. Notably, HCV seroprevalence was significantly higher in Lahore than in Quetta in our population. High HCV prevalence among injection drug users can foreshadow future epidemics of HIV infection, as was detected recently in Estonia [26].

Despite such high HCV prevalence, less than 20% of our sample was aware of HCV compared to 85% who were aware of HIV, underscoring the need for expanded education about HCV. To our knowledge, no formal country-wide guidelines for HCV education have yet been developed in Pakistan, although a new hepatitis B virus and HCV awareness campaign was recently launched in Peshawar [27]. There was no difference in HCV prevalence between those who were and were not aware of HCV. It should be noted that although HIV awareness was relatively high, the majority of the study population continued to be actively engaged in high-risk behaviors, suggesting that individuals may not fully understand the mechanisms of disease transmission or health consequences. Of concern, only 6% study participants returned to receive their HIV and HCV test results, underscoring the need for HIV and HCV educational prevention programs to focus on reducing the stigma of HIV and HCV and engaging individuals in raising awareness of one's HIV and HCV serostatus and associated health risks.

As expected, injecting behaviors, such as duration of injection and the intentional act of drawing blood into the syringe while injecting (i.e., 'jerking'), were strongly associated with HCV seropositivity. It is unknown how the practice of jerking was initiated in Pakistani drug users. Anecdotal reports from treatment providers in Pakistan indicate that IDUs prefer this practice because it gives injectors a 'better high'. Similar injection behaviors such as booting and registering are common in North America and in Europe [19-21]. Interestingly, similar behaviors have recently emerged in other continents; a report from Tanzania revealed a newly-observed needle sharing practice common among female sex workers called "flashblood," in which a female heroin injector will draw several milliliters of her blood into a syringe and pass it to another woman for her to inject into her vein. This practice is believed to prevent symptoms of heroin withdrawal [28].

Although the practice of jerking alone would not appear to confer an elevated risk of infection in the absence of sharing injection equipment, further analysis revealed that jerking was significantly associated with needle sharing in both Quetta and Lahore. This may explain why needle sharing was not independently associated with HCV seropositivity after controlling for other risk factors and suggests that needle sharing behaviors may be underreported. Previous studies have reported that IDUs underestimate the extent to which they engage in sharing of injection paraphernalia [29,30].

Among IDUs, HCV prevalence was significantly higher among IDUs in Lahore who had been injecting drugs for two or more years and is consistent with findings from previous studies [31]. The fact that HCV prevalence was higher in Lahore than in Quetta is likely explained by the fact that injectors from Lahore had been injecting for significantly longer. Most drug users in Lahore injected drugs rather than chased the dragon, while the opposite was true in Quetta. Quetta is situated very close to Afghanistan, the world's largest producer of heroin [32]; due to its proximity to the border and relatively easy access to heroin supplies, heroin use in Quetta mainly consisted of chasing and smoking. Towards the end of its rule in 2001, the Taliban government in Afghanistan prohibited the opium trade and further interruptions in the drug trade as a result of the U.S-Afghan war led to lower availability of heroin in the surrounding area (e.g., Quetta) and is thought to have facilitated the recent trend in switching from heroin chasing to injection of pharmaceutical opiates [33].

Other behaviors unrelated to drug use were also independently associated with HCV seropositivity. IDUs from Quetta who had ever received a barber shave had a marginally higher odds of HCV seropositivity, consistent with other studies in non-drug using populations in both developed and developing nations [34-38]. Barbering in makeshift stalls or on the street is common in Pakistan and is often conducted under unhygienic conditions. The lifetime prevalence of barber shaving was very high in our study population, resulting in a wide confidence interval. Although this association should be interpreted with caution, barbers should be advised to ensure that their shaving equipment is properly sterilized, and communities should be educated about the potential risks of acquiring HIV/HCV infections through these means.

Having ever worked outside of Pakistan was inversely associated with HCV seropositivity. Although this association was statistically significant in Quetta only, a protective effect was also seen among IDUs in Lahore. The protective effect of working abroad may be explained by a shorter exposure to injection-related risk behaviors while working abroad and having greater economic resources. Previous reports suggested that HIV had been imported into Pakistan from migrant workers who had gone abroad, mainly to the Gulf States for temporary work, returning home unknowingly infected with HIV [39,40]. Since HIV was non-existent in our sample, we found no support for this hypothesis in relation to HCV infection. Self-reported homelessness was also associated with HCV seropositivity but is most likely a marker for low socioeconomic status or other risk behaviors not fully assessed in this study.

Limitations of our analysis included the fact that using a sample of health-seeking individuals attending a harm reduction clinic may underestimate the true risk of HIV and HCV seropositivity among drug users in Pakistan. Also, generalizability of these results to other drug users in Pakistan is unclear, especially given the different patterns of drug use between the two cities. Future prevention programs should be tailored to the site-specific populations in order to be most effective. In addition, stratification by site reduced the sample size in each analysis and reduced the power to detect statistically significant differences, particularly in Quetta.

Despite these limitations, these data provide a useful risk profile that can be used to develop tailored prevention programs for these high risk populations. Additional interventions to prevent HIV/HCV transmission should also include increasing the availability of sterile needles through needle exchange programs or pharmacies and expanding drug treatment to prevent or curb injection behaviors. Currently, opiate substitution therapies are not legally available as a form of drug abuse treatment in Pakistan (T. Zafar, personal communication, 2005); introduction of methadone maintenance and other substitution therapies in Pakistan could help prevent HIV transmission by reducing injection risks among IDUs, as has recently been endorsed by the United Nations and the World Health Organization [41]. Moreover, newly-initiated injection drug users should be targeted for these education and prevention programs to prevent the further spread of HCV infection.

## Conclusion

This study suggests that conditions exist for a potential HIV outbreak to occur and for the continued transmission of HCV in the drug using population. The recent HIV outbreak in the southern city of Karachi foreshadows potentially explosive HIV outbreaks in other major urban areas in Pakistan given the high-risk behaviors we observed, as has been seen in many other countries with similar risk profiles. Our data suggest there is a very short window of opportunity to prevent a potential HIV epidemic among drug users in eastern and western Pakistan, and site-specific interventions should be developed and implemented immediately.

## Competing interests

The author(s) declare that they have no competing interests.

## Authors' contributions

IK and SAS conceived of the study, conducted data analysis and wrote the first drafts of the manuscript. NG, MA and DLT provided statistical analytic support, cultural interpretation, and clinical relevance and interpretation. SU and TZ managed the study, collected the data in Pakistan, and provided cultural interpretation. All authors participated in the interpretation of the data and in the final review of the manuscript.
